# Supplementary material for: Implementation of the Infant-Toddler Checklist in Swedish child health services at 18 months: an observational study
Source: BMJ Paediatr Open. 2024 Mar 25;8(1):e002406. doi: 10.1136/bmjpo-2023-002406 (PMC10966819; doi:10.1136/bmjpo-2023-002406)
Supplement: Author's manuscript [file bmjpo-2023-002406.draft_revisions.pdf]

BMJ Paediatrics Open

BMJ Paediatrics Open is committed to open peer review. As part of this commitment we make the peer review history of every article we publish publicly available.

When an article is published we post the peer reviewers' comments and the authors' responses online. We also post the versions of the paper that were used during peer review. These are the versions that the peer review comments apply to.

The versions of the paper that follow are the versions that were submitted during the peer review process. They are not the versions of record or the final published versions. They should not be cited or distributed as the published version of this manuscript.

BMJ Paediatrics Open is an open access journal and the full, final, typeset and author-corrected version of record of the manuscript is available on our site with no access controls, subscription charges or pay-per-view fees (http://bmjpaedsopen.bmj.com).

If you have any questions on BMJ Paediatrics Open's open peer review process please email
info.bmjpo@bmj.com

BMJ Paediatrics Open

A natural experiment evaluating the implementation of the Infant-Toddler Checklist in Swedish child health services

Journal:	*BMJ Paediatrics Open*
Manuscript ID	bmjpo-2023-002406
Article Type:	Original research
Date Submitted by the Author:	24-Nov-2023
Complete List of Authors:	Dahlberg, Anton; Uppsala University, Child Health and Parenting (CHAP), Department of Public Health and Caring Sciences<br>Levin, Anna; Uppsala University, Child Health and Parenting (CHAP), Department of Public Health and Caring Sciences<br>Fäldt, Anna; Uppsala Universitet, Dept of Public Health and Caring Sciences
Keywords:	Screening, Infant

SCHOLARONE™
Manuscripts

I, the Submitting Author has the right to grant and does grant on behalf of all authors of the Work (as defined in the below author licence), an exclusive licence and/or a non-exclusive licence for contributions from authors who are: i) UK Crown employees; ii) where BMJ has agreed a CC-BY licence shall apply, and/or iii) in accordance with the terms applicable for US Federal Government officers or employees acting as part of their official duties; on a worldwide, perpetual, irrevocable, royalty-free basis to BMJ Publishing Group Ltd ("BMJ") its licensees and where the relevant Journal is co-owned by BMJ to the co-owners of the Journal, to publish the Work in this journal and any other BMJ products and to exploit all rights, as set out in our licence.

The Submitting Author accepts and understands that any supply made under these terms is made by BMJ to the Submitting Author unless you are acting as an employee on behalf of your employer or a postgraduate student of an affiliated institution which is paying any applicable article publishing charge ("APC") for Open Access articles. Where the Submitting Author wishes to make the Work available on an Open Access basis (and intends to pay the relevant APC), the terms of reuse of such Open Access shall be governed by a Creative Commons licence – details of these licences and which Creative Commons licence will apply to this Work are set out in our licence referred to above.

Other than as permitted in any relevant BMJ Author's Self Archiving Policies, I confirm this Work has not been accepted for publication elsewhere, is not being considered for publication elsewhere and does not duplicate material already published. I confirm all authors consent to publication of this Work and authorise the granting of this licence.

Confidential: For Review Only

1

A Natural Experiment Evaluating the Implementation of The Infant-Toddler Checklist in Swedish Child Health Services

Anton DAHLBERG[a], Anna LEVIN[a], Anna FÄLDT[a]*

*Corresponding author

[a]Child Health and Parenting (CHAP), Department of Public Health and Caring Sciences, Uppsala University, Box 564, 751 22 UPPSALA Sweden

Anna Fäldt

anna.faldt@uu.se Telephone: +46 76 776 77 55

orcid: 0000-0001-7257-8758

Anton Dahlberg

Anton.dahlberg@uu.se Telephone: +46 18-471 6574

orcid:0000-0003-0136-8862

Anna Levin

Anna.levin@gotland.se Telephone: +46 498-26 85 01

Competing interest declaration:

Author 2, Anna Levin, was involved in the planning and implementing of the ITC in the region of Gotland.

Confidential: For Review Only

2

WHAT IS KNOWN ABOUT THE SUBJECT:

- Children with communication disorders should receive timely interventions
- Implementation is difficult

WHAT THIS STUDY ADDS:

- ITC can be implemented relatively quickly.

HOW THIS STUDY MIGHT AFFECT PRACTICE

- Stakeholders at the child health services should consider implementing a scientifically evaluated screening method at 18 months to enable early interventions in settings where interventions are available.

Confidential: For Review Only

ABSTRACT

Background and objectives:

Communication and language disorders are common conditions that emerge early and negatively impact the lifespan. Early identification and interventions can affect the child's language development and reduce the risk of secondary negative impact. There is a need to introduce more accurate screening methods.

Using the RE-AIM implementation framework in a Swedish county, this study assessed whether children with suspected language and communication difficulties were identified and referred earlier after the introduction of the screening method Infant-Toddler Checklist at the 18-month visit at the child health services.

Methods:

All data were collected from the medical journal records for children who visited the child health services at 17–22 months. Data collection took place during two periods: before the introduction of the Infant-Toddler Checklist (1,717 children) and after the introduction of the Infant-Toddler Checklist (916 children).

Results:

Children were identified and referred to speech and language therapy at the same proportion. However, they were referred earlier after the introduction of the Infant-Toddler Checklist compared with before its introduction, with a mean referral age at 24 and 29 months, respectively. The adoption rate at setting-level was 100%. The implementation process took about three months until an acceptable ratio of children were screened with the Infant-Toddler Checklist (94–100%). The use of the Infant-Toddler Checklist was sustained over time.

Conclusion

The implementation outcomes were positively affected through staff training, improvements in the medical record system, close monitoring of the implementation and written procedures. The Infant-Toddler Checklist can be an effective instrument for identifying children earlier, thus enable timely interventions.

KEYWORDS:

Implementation

RE-AIM

Screening

Child Health Services

Early identification

Language disorders

Confidential: For Review Only

INTRODUCTION

Communication and language are the basis of human interaction and are essential for development, learning and health. Language and communication difficulties can affect a child's whole life and lead to a lower quality of life, including mental health problems, school difficulties and social exclusion.[1 2]

Language and communication difficulties are common affecting around 10% of children in the Western world.[3] There is also considerable overlap between language and communication difficulties and other neurodevelopmental conditions.[3-5]

Early interventions for language and communication difficulties affect children's language development and are hypothesised to reduce the risk of secondary negative impacts such as disturbance in emotional and social adaptation. [6-10] However, screening for language disorder is not recommended for children younger than four.[11 12] In contrast, early screening is recommended for autism, where language or communication difficulties can be the first symptom.[11 13] To enable timely interventions, there is a need for validated and evidence-based screening instruments.[14] Several methods are available to identify children with communication and language difficulties early,[15 16] but implementation in child health services (CHS) is still in its infancy. The Swedish CHS is regulated by a National programme[17] and reach almost all children, aiming to promote health and monitor children's development,[18] making them an excellent arena for early language screening. Children's communication and language are monitored at 18 months by asking parents if the child speaks at least 8–10 words and understands more than 8–10 words. This practice has been found to have a low sensitivity;[19] thus, more accurate screening methods are needed.

One method for identifying children with language and communication difficulties is the Communication and Symbolic Behavior Scales Developmental Profile Infant-Toddler Checklist (ITC).[20] The ITC detects a range of developmental concerns, including language delay, global developmental delay and autism.[16 21 22] When the ITC was used in primary care children with developmental delay were identified with high specificity, high negative predictive value and low false positive rate.[23] ITC used at the 18-month visits in a Swedish CHS showed a sensitivity of 0.85 and specificity of 0.59 which increased to 0.88 and 0.63, respectively when the ITC was combined with the nurse's assessment.[24] Further, nurses has highlighted the usability and relative advantages of using the ITC in CHS.[25]

Confidential: For Review Only

5

Setting

The ITC screening was introduced at the 18-month visit for children born from January 2019 in Region of Gotland, the smallest county in Sweden, with 500–600 children born annually. The CHS is divided into three centres in different geographical areas with different socioeconomic demographics.[26] The nurses were supported in implementation, consultation and education by a central CHS unit consisting of a nurse, a paediatrician and a psychologist.

Implementation strategies

Introducing new approaches[27] and transferring methods tested in research studies into everyday clinical practice is complex and takes time. [27] Applying an implementation framework when assessing an intervention allows one to investigate the process'*s how* and *why*, which can facilitate identifying and understanding effective components and barriers in the implementation process. [28] This, in turn, can form a rationale for future implementation.

In this study, the RE-AIM framework was applied to describe the implementation of the ITC in CHS.[29] The RE-AIM offers a structured way of describing, employing and analysing implementation processes at five key dimensions: the *reach* of the target population, *effectiveness*, *adoption*, *implementation* and *maintenance* of the implemented method.[30]

Research questions

1. What proportion of eligible children were screened with the ITC after its implementation? (Reach)
2. Did the implementation of the ITC lead to earlier referrals to Speech and language therapy (SLT) for children with suspected language or communication difficulties? (Effectiveness)
3. Was the ITC adopted equally across all centres? (Adoption)
4. What actions were taken to facilitate the implementation of the ITC during the study period? (Implementation)
5. Was the implementation of the ITC sustained over time, and if so, how? (Maintenance)

METHODS

Screening instrument

The ITC consists of 24 multiple-choice questions in three subscales: social, speech, and symbolic, and a total score.[20] If the social or symbolic subscales or the total score are below a cut-off value (fail) and thus indicate a

Confidential: For Review Only

possible divergent communication development, the child should be referred to a specialist. If only the speech scale indicates concern, the child should be reassessed with the ITC after three months.[16]

Data collection

Regional documentation regarding implementation strategies was collected from the CHS unit. Pseudonymised data, including child age at screening, sex, screening method and results, referral and age at referral to SLT and centre, were collected via the medical records system.

As the ITC-specific item-selection keywords were not applied in the medical records system during the first months after the implementation, both data based on free text entries and ITC-specific item-selections were processed. The collected ITC free-form text entries contained information on completing the ITC screening (*ITC completed*/*ITC not completed*) and potential concern for the child's communication development (*concern/no concern*). Stated reasons for not referring children who failed the screening were collected.

Research Ethics Approval

This study was conducted according to the ethical guidelines described in the Helsinki Declaration and was approved by the Swedish Ethical Review Board (Dnr 2021-04737).

Study population

To answer the different research questions, two samples were used. One subsample included all children receiving their 18-month CHS visit (17–22 months) between May 2016 and April 2022 (n=2,633 children, girls 48,8 %). The intervention group (n=916, 48,7% girls), henceforth the ITC group, was exposed to ITC (June 2020–April 2022), while the control group (n=1717, 48,8% girls) received care as usual (May 2016–June 2020).

To answer the question regarding the effectiveness of the ITC in terms of early referral (before 25 months of age), a subsample was used, including children who had reached at least 30 months of age (*n*=2163). The control group consisted of 1717 children (48,8% girls), and the ITC group of 446 children (46,6% girls). The study design can be viewed as a natural experiment.[31] As this study was based on register data, no involvement of patients were possible.

Analysis

Reach was explored through the ratio between the screened children and the total number of children in the ITC group. The effectiveness was explored through descriptive statistics with the number of children who failed the screening, and referrals. To analyse the differences in early referral rates (effectiveness), the Chi-squared test

Confidential: For Review Only

with Yates' continuity correction was used. Effect size was measured through odds ratio. Gender differences in early referral were assessed through Chi-squared test. Data were prepared and analysed using the statistical software R and SPSS.

RESULTS

Following the structure of the implementation framework, results are presented for each domain (Figure 1).

	Reach	Effectiveness	Adoption	Implementation	Maintenance
**Question**	What proportion of eligible children were screened with the ITC after its implementation?	Did the implementation of the ITC lead to earlier referrals for children with suspected language or communication difficulties?	Was the ITC adopted equally across all CHCs?	What actions were taken to facilitate the implementation of the ITC during the study period?	Was the implementation of the ITC sustained over time and, if so, how?
**Result**	93% screening rate	Significant difference in referrals between ITC and previous method (OR = 2.07)<br>No significant gender differences	All centres adopted the method (100% adoption rate)	• Training session<br>• Close collaboration<br>• Written procedure<br>• Automatic procedure<br>• Modified medical record procedure	Time to full implementation: 3 months<br>Implementation maintained over full study period<br>Brief drop in screening rate during first 6 months

Reach

Of the 916 children in the ITC group, 854 were screened with the ITC, representing a reach of 93%. For 11 children, the ITC was reported as failing to be returned to the CHS. For the remaining 51 children, no reasons were given for the children not being screened.

Effectiveness

Of the 854 children screened with the ITC, 120 children (14%) failed the screening, of which 51 children failed on speech and thus would be rescreened with the ITC three months later. For 18 children, screening with the ITC had been performed on two occasions, failing on both occasions. Seventy-five children who failed the screening were not referred early to SLT. Thirty-eight of these children had failed on the *speech* subscale of the ITC only; therefore, holding off on referral was correct.[20] For nine children, it was indicated that the parents expressed no concern or declined the referral offer. The nurses stated no concerns regarding the child's communication development for nine children. Of the remaining 18 children who failed the screening, no reason was given for not sending referrals.

Analysing the subsample of children aged at least 30 months, a significant relationship between screening method and early referral was detected, where the ITC was associated with more early referrals (χ^2 *(n=2,164) =11.0, p*<.001). The odds for early referral were twice as high for children screened with the ITC (OR=2.07, 95% CI [1.34, 3.14]). In the control group, 16% of the referred children were girls, while this rate was 23% in

Confidential: For Review Only

the ITC group. However, no significant differences were found between the two groups regarding gender differences in referral.

Adoption

All centres implemented ITC, resulting in a 100% adoption rate at setting level, which remained unchanged during the study period. At the centre level, there was variation in the proportion of children screened (range 92–97%) with the highest adherence in the most socio-economically challenged area.

Implementation and implementation strategies

The implementation was performed in collaboration between the central CHS unit and the SLT services. Prior to the implementation of the ITC, staff attended training sessions and workshops on parent-child relationships, early communication development, the importance of early intervention and how to perform the ITC. A written procedure was developed, including referrals and re-screenings. Shortly after implementing the ITC, a medical record system was modified to include an ITC-specific template with fixed response options for record entry. Before the modification, the nurses entered the results of the ITC screening in free-form text fields into the medical record. Further, the medical record system automatically included the ITC with the invitation letter to the 18-month visit.

Maintenance

During the study, the CHS unit, mainly through a designated project manager, kept close contact with the nurses, offering support feedback and monitoring the implementation process. The screening procedure instructions were continuously updated and improved throughout the implementation. The use of the ITC was sustained over time during the entire study period and is, as of 2023, still part of routine practice across the county.

The consistency of implementation varied in the early phase of implementation, as the proportion of children screened was initially low: in the first three months, 20% of the children were not screened. Thereafter, the rate of screened children varied between 94% and 100%.

In January 2022, the rate of screened children dropped to 87%. This coincided with the start of a short-term employment of a substitute nurse. By March 2022, the rate of screened children had increased to 94%.

DISCUSSION

Confidential: For Review Only

Introducing new procedures and methods to everyday clinical practice is often complicated and challenging.[32] It is, therefore, essential to monitor the implementation processes. This study aimed to enhance knowledge regarding implementing the ITC through RE-AIM. [29 30]

The reach of the ITC was high; 93% of children who visited the CHS since the introduction were screened with the ITC. One risk of using screening instruments is that children are not identified equally across demographics.[33] No individual socio-demographic data were available in this study. Therefore, it is impossible to conclude whether children are screened and identified equally. However, the three centres were located in areas with diverse socioeconomic makeup, and results suggested that adoption was highest in the most socio-economically challenged areas.[26]

There was a large difference in referrals between girls and boys using both methods, although the difference tended to be smaller after the introduction of ITC. Language and communication difficulties have been presumed to be more common in boys than in girls. Still, the differences are often minor and non-significant.[3] On the other hand, the boy-girl ratio for receiving intervention is significant,[34] which can result from differences in identification. It is essential to study reasons for gender differences in identification and referral in future studies.

Implementation took place in inter-organisational and inter-professional collaboration. This presumably positively affected the implementation and facilitated planning and communication in the short and long term. As anticipated, the implementation had a start-up period of three months before almost all children were screened with the ITC, a period considered short compared with other implementations.[35] Preparations for the implementation included workshops and training for the nurses. This can be seen as a beneficial factor to the high adoption rate and reach and an important part of the implementation, as the nurses were relatively well prepared for the implementation process.

The central CHS unit included a designated project manager, who followed the implementation of the ITC closely. The continuous improvement of the written procedures and routines may have facilitated the implementation, created reassurance for the nurses and reduced the risk of incorrect practice. The automated printing and including the ITC with the invitation letter to the parents reduced the risk of failing to send out the questionnaires. Further, the revised medical journal template reminded the nurses and facilitated correct record-keeping.

Confidential: For Review Only

10

The importance of ongoing training and supervision to facilitate maintenance is highlighted by the decrease in the proportion of children screened when new staff are recruited.

Some children who failed the ITC screening were not referred to SLT although they were screened positive with the ITC. While this was mainly explained through best practice or parents declining referral, for some children, no reason was given for not sending a referral. Adherence to referral guidelines can be low and might depend on family demographics.[36] Even though ITC has been described as giving parents insight into their child's development and facilitated referrals, it can be difficult to motivate parents referral to SLT at 18 months.[25] Professionals sometimes hold off on referral, even when parents express concern.[37] The psychometric properties of the ITC could be a possible contributing factor to the lower referral rate, as the specificity of the ITC is somewhat low when used at 18 months.[24] This is a relatively common challenge in screening at younger ages. Screening may need to be supplemented with the nurse's clinical observation and a dialogue with the parent.

The goal of implementing the ITC was to ensure that children needing support in their communication and language development would be identified earlier and referred to specialist services such as SLT. For early referrals to be meaningful, interventions must be available in conjunction with the referrals. In the Region of Gotland, such a timely intervention is available, which is not the case in all of Sweden, where the waiting times for SLT interventions can be years long.[38]

One basic principle of screening is that only children needing interventions should be identified,[39] which is especially important when resources are scarce. This puts extra demands on the use of clinically valid screening instruments. The ITC could be one such instrument, as the analyses show that the referral rate is equal before and after the implementation of the ITC but that the referrals occur when the children are younger. Future studies should assess how ITC affects the care process for children how the ITC relates to later language screenings.

CONCLUSIONS

The implementation of the ITC was associated with high reach, earlier referrals, complete adoption, and sustained maintenance over time. Thus, the ITC implementation process was successful in relation to the RE-AIM domains. The successful implementation was facilitated through training, improvements in the medical record system, close monitoring of the implementation and written procedures that may have contributed to the outcome.

Confidential: For Review Only

The ITC can be used for early detection of language and communication difficulties and hence help families receive timely interventions. Depending on context, a similar implementation could be beneficial, as there is a general need for earlier screening of language and communication difficulties to enable timely interventions.

CONTRIBUTORS

AF, AD, AL: conceptualised, designed the study and drafted the original manuscript. AF and AD: data analysis, AL: performed electronic health record data extraction. All authors reviewed and approved the final manuscript as submitted. AF willingly accepted the role of guarantor for this study.

FUNDING INFORMATION

The study was funded by Länsförsäkringar Gotland

ACKNOWLEDGEMENTS

The authors would like to thank the child health service in the Region of Gotland for their contribution.

Confidential: For Review Only

REFERENCES

1. McCormack J, McLeod S, McAllister L, Harrison LJ. A systematic review of the association between childhood speech impairment and participation across the lifespan. *Int J Speech Lang Pathol* 2009;11(2):155-70. doi: 10.1080/17549500802676859
2. den Bedem NP, Dockrell JE, Alphen PM, et al. Depressive symptoms and emotion regulation strategies in children with and without developmental language disorder: a longitudinal study. *Int J Lang Commun Disord* 2018;53(6):1110-23. doi: 10.1111/1460-6984.12423
3. Norbury CF, Gooch D, Wray C, et al. The impact of nonverbal ability on prevalence and clinical presentation of language disorder: evidence from a population study. *J Child Psychol* 2016;57(11):1247-57. doi: 10.1111/jcpp.12573
4. Sciberras E, Mueller KL, Efron D, et al. Language problems in children with ADHD: A community-based study. *Pediatrics (Evanston)* 2014;133(5):793-800. doi: 10.1542/peds.2013-3355
5. McGrath LM, Hutaff-Lee C, Scott A, et al. Children with Comorbid Speech Sound Disorder and Specific Language Impairment are at Increased Risk for Attention-Deficit/Hyperactivity Disorder. *J Abnorm Child Psychol* 2007;36(2):151-63. doi: 10.1007/s10802-007-9166-8
6. Warren Z, McPheeters ML, Sathe N, et al. A Systematic Review of Early Intensive Intervention for Autism Spectrum Disorders. *Pediatrics* 2011;127(5):e1303.
7. Roberts MY, Curtis PR, Sone BJ, Hampton LH. Association of Parent Training With Child Language Development A Systematic Review and Meta-analysis. *JAMA PEDIATRICS* 2019;173(7):671-80. doi: 10.1001/jamapediatrics.2019.1197
8. Dubin AH, Lieberman-Betz RG. Naturalistic Interventions to Improve Prelinguistic Communication for Children with Autism Spectrum Disorder: a Systematic Review. *Review Journal of Autism and Developmental Disorders* 2020;7(2):151-67. doi: 10.1007/s40489-019-00184-9
9. Novak I, Morgan C. Chapter 23 - High-risk follow-up: Early intervention and rehabilitation. In: de Vries LS, Glass HC, eds. Handb Clin Neurol: Elsevier 2019:483-510.
10. Rinaldi S, Caselli MC, Cofelice V, et al. Efficacy of the Treatment of Developmental Language Disorder: A Systematic Review. *Brain Sci* 2021;11(3):407. doi: 10.3390/brainsci11030407
11. Jullien S. Screening for language and speech delay in children under five years. *BMC Pediatr* 2021;21(Suppl 1):362-62. doi: 10.1186/s12887-021-02817-7
12. Bishop DVM, Snowling MJ, Thompson PA, et al. CATALISE: A Multinational and Multidisciplinary Delphi Consensus Study. Identifying Language Impairments in Children. *PLoS One* 2016;11(7):e0158753. doi: 10.1371/journal.pone.0158753
13. Jullien S. Screening for autistic spectrum disorder in early childhood. *BMC Pediatr* 2021;21(1):349. doi: 10.1186/s12887-021-02700-5
14. Horiuchi S, Nomura O, Curry L, et al. “Caregivers’ awareness matters”: Call for a child-centered approach to early detection of autism spectrum disorder through well-child visits in Japan. *Child Care Health Dev* 2023
15. Sim F, Thompson L, Marryat L, et al. Predictive validity of preschool screening tools for language and behavioural difficulties: A PRISMA systematic review. *PLoS One* 2019;14(2):e0211409. doi: 10.1371/journal.pone.0211409

Confidential: For Review Only

16. Wetherby AM, Brosnan-Maddox S, Peace V, Newton L. Validation of the Infant—Toddler Checklist as a broadband screener for autism spectrum disorders from 9 to 24 months of age. *Autism* 2008;12(5):487-511.
17. Tell J. Swedish Child health services: Inera; 2019 [cited 2020 29 September]. Available from: https://www.rikshandboken-bhv.se/metoder--riktlinjer/informationsmaterial/swedish-child-health-services/ accessed September 29 2020.
18. Wallby T, Hjern A. Child health care uptake among low-income and immigrant families in a Swedish county. *Acta Paediatr* 2011;100(11):1495-503. doi: 10.1111/j.1651-2227.2011.02344.x
19. Westerlund M, Berglund E, Eriksson M. Can Severely Language Delayed 3-Year-Olds Be Identified at 18 Months? Evaluation of a Screening Version of the MacArthur-Bates Communicative Development Inventories. *J Speech Lang Hear Res* 2006;49(2):237-47. doi: 10.1044/1092-4388(2006/020)
20. Wetherby AM, Prizant BM. Communication and Symbolic Behavior Scales Developmental Profile, First Normed Edition: Brookes Publishing Co 2002.
21. Wetherby AM, Allen L, Cleary J, et al. Validity and reliability of the communication and symbolic behavior scales developmental profile with very young children. *J Speech Lang Hear Res* 2002;45(6):1202-18.
22. Wetherby AM, Goldstein H, Cleary J, et al. Early identification of children with communication disorders: concurrent and predictive validity of the CSBS Developmental Profile. *Infants & Young Children: An Interdisciplinary Journal of Early Childhood Intervention* 2003;16(2):161-74 14p.
23. Borkhoff CM, Atalla M, Bayoumi I, et al. Predictive validity of the Infant Toddler Checklist in primary care at the 18-month visit and developmental diagnosis at 3–5 years: a prospective cohort study. *BMJ Paediatrics Open* 2022;6(1):e001524. doi: 10.1136/bmjpo-2022-001524
24. Fäldt A, Fabian H, Dahlberg A, et al. Infant-Toddler Checklist identifies 18-month-old children with communication difficulties in the Swedish child healthcare setting. *Acta Paediatr* 2021;110(5):1505-12. doi: 10.1111/apa.15696
25. Fäldt A, Nordlund H, Holmqvist U, et al. Nurses' experiences of screening for communication difficulties at 18 months of age. *Acta Paediatr* 2019;108(4):662-69. doi: 10.1111/apa.14557
26. DEMOS. Segregationsbarometern [Segregation barometer] Huddinge, Sweden2022 [Available from: https://segregationsbarometern.delmos.se/kommun/gotland/ accessed 2022-09-07.
27. Woolf SH. The meaning of translational research and why it matters. *Jama* 2008;299(2):211-13. doi: 10.1001/jama.2007.26
28. Rubenstein LV, Pugh J. Strategies for promoting organizational and practice change by advancing implementation research. *Journal of General Internal Medicine* 2006;21(2):S58. doi: 10.1111/j.1525-1497.2006.00364.x
29. Glasgow RE, Vogt TM, Boles SM. Evaluating the public health impact of health promotion interventions: the RE-AIM framework. *American journal of public health (1971)* 1999;89(9):1322-27. doi: 10.2105/AJPH.89.9.1322
30. Glasgow RE, Harden SM, Gaglio B, et al. RE-AIM Planning and Evaluation Framework: Adapting to New Science and Practice With a 20-Year Review. *Frontiers in Public Health* 2019;7(64) doi: 10.3389/fpubh.2019.00064
31. Dunning T. Natural experiments in the social sciences : a design-based approach. Cambridge: Cambridge University Press 2012.

Confidential: For Review Only

32. Damschroder LJ, Aron DC, Keith RE, et al. Fostering implementation of health services research findings into practice: a consolidated framework for advancing implementation science. *Implementation science : IS* 2009;4(1):50-50. doi: 10.1186/1748-5908-4-50
33. Guthrie W, Wallis K, Bennett A, et al. Accuracy of Autism Screening in a Large Pediatric Network. *Pediatrics* 2019;144(4):e20183963. doi: 10.1542/peds.2018-3963
34. Morgan PL, Farkas G, Hillemeier MM, et al. Cross-Cohort Evidence of Disparities in Service Receipt for Speech or Language Impairments. *Except Child* 2017;84(1):27-41. doi: 10.1177/0014402917718341
35. Morris ZS, Wooding S, Grant J. The answer is 17 years, what is the question: understanding time lags in translational research. *J R Soc Med* 2011;104(12):510-20. doi: 10.1258/jrsm.2011.110180
36. Wallis KE, Guthrie W, Bennett AE, et al. Adherence to screening and referral guidelines for autism spectrum disorder in toddlers in pediatric primary care. *PLoS One* 2020;15(5):e0232335. doi: 10.1371/journal.pone.0232335
37. Weitlauf AS, Vehorn A, Miceli A, et al. Black Families' Experiences of Developmental Screening: Review of Well-Child Visits to Inform Enhanced Autism Spectrum Disorder Risk Assessment. *J Dev Behav Pediatr* 2022;43(9):503-10. doi: 10.1097/DBP.0000000000001129
38. Marjomaa J. Lång kö till logoped för barn i Västra Götaland [Long waiting time to get SLT service for children in Västra Götaland]. SVT Väst. Göteborg, 2021.
39. Wilson J, Jungner G. Principles and practice of screening for disease. Geneva: WHO, 1968:27.

Confidential: For Review Only

BMJ Paediatrics Open

Implementation of the Infant-Toddler Checklist in Swedish child health services at 18 months: an observational study

Journal:	*BMJ Paediatrics Open*
Manuscript ID	bmjpo-2023-002406.R1
Article Type:	Original research
Date Submitted by the Author:	14-Feb-2024
Complete List of Authors:	Dahlberg, Anton; Uppsala University, Child Health and Parenting (CHAP), Department of Public Health and Caring Sciences<br>Levin, Anna; Uppsala University, Child Health and Parenting (CHAP), Department of Public Health and Caring Sciences<br>Fäldt, Anna; Uppsala Universitet, Dept of Public Health and Caring Sciences
Keywords:	Screening, Infant

SCHOLARONE™
Manuscripts

BMJ

I, the Submitting Author has the right to grant and does grant on behalf of all authors of the Work (as defined in the below author licence), an exclusive licence and/or a non-exclusive licence for contributions from authors who are: i) UK Crown employees; ii) where BMJ has agreed a CC-BY licence shall apply, and/or iii) in accordance with the terms applicable for US Federal Government officers or employees acting as part of their official duties; on a worldwide, perpetual, irrevocable, royalty-free basis to BMJ Publishing Group Ltd ("BMJ") its licensees and where the relevant Journal is co-owned by BMJ to the co-owners of the Journal, to publish the Work in this journal and any other BMJ products and to exploit all rights, as set out in our licence.

The Submitting Author accepts and understands that any supply made under these terms is made by BMJ to the Submitting Author unless you are acting as an employee on behalf of your employer or a postgraduate student of an affiliated institution which is paying any applicable article publishing charge ("APC") for Open Access articles. Where the Submitting Author wishes to make the Work available on an Open Access basis (and intends to pay the relevant APC), the terms of reuse of such Open Access shall be governed by a Creative Commons licence – details of these licences and which Creative Commons licence will apply to this Work are set out in our licence referred to above.

Other than as permitted in any relevant BMJ Author's Self Archiving Policies, I confirm this Work has not been accepted for publication elsewhere, is not being considered for publication elsewhere and does not duplicate material already published. I confirm all authors consent to publication of this Work and authorise the granting of this licence.

Confidential: For Review Only

Implementation of the Infant-Toddler Checklist in Swedish child health services at 18 months: an observational study

Anton DAHLBERG[a], Anna LEVIN[a], Anna Erica FÄLDT[a]*

*Corresponding author

[a]Child Health and Parenting (CHAP), Department of Public Health and Caring Sciences, Uppsala University, Box 564, 751 22 UPPSALA Sweden

Anna Fäldt

anna.faldt@uu.se Telephone: +46 76 776 77 55

orcid: 0000-0001-7257-8758

Anton Dahlberg

Anton.dahlberg@uu.se Telephone: +46 18-471 6574

orcid:0000-0003-0136-8862

Anna Levin

Anna.levin@gotland.se Telephone: +46 498-26 85 01

Competing interest declaration:

Author 2, Anna Levin, was involved in the planning and implementing of the ITC in the region of Gotland.

Confidential: For Review Only

ABSTRACT

Background: Communication and language disorders are common conditions that emerge early and negatively impact quality of life across the life course. Early identification may be facilitated using a validated screening tool such as the Infant Toddler Checklist (ITC). We introduced the ITC at the 18-month visit at child health services in a Swedish county. Using the RE-AIM implementation framework, this study assessed the implementation of the ITC according to five key dimensions: reach, effectiveness, adoption, implementation, and maintenance.

Methods: This observational study used medical records at child health services as data source. Data were collected from children who visited a child health nurse at 17–22 months. The sample included 2633 children with a mean age of 17.8 months, 1717 in the pre-implementation group and 916 in the post-implementation group. We calculated ITC completion rate (reach) and use at each site (adoption). We compared rates of referral to speech and language therapy (effectiveness) before and after implementation of the ITC using Odds Ratio (OR) and 95% Confidence Intervals (CI). We described actions to facilitate implementation and maintenance of ITC screening over time.

Results: The overall screening rate was 93% (reach) which increased from 80% initially to 94% at the end of the 2-year period (maintenance). All centres used the ITC (adoption). The ITC screen positive rate was 14%. Of children who had reached at least 24 months (n=2367), referral rate was 0.4% pre-implementation versus 6.9% post-implementation (OR = 18.17, 95% CI [8.15, 40.51], $p < 0.001$) (effectiveness). Implementation strategies included training sessions, collaboration, written and automatic procedures and modifications to the medical records system.

Conclusion: The implementation of the ITC was associated with high reach, higher referral rate, complete adoption, and sustained maintenance over time.

KEY MESSAGE

What is known about the subject:

- Early language assessment is essential for offering timely interventions to children with communication disorders.
- Implementing and maintaining new screening procedures in healthcare is difficult and research on this is scarce.

Confidential: For Review Only

What this study adds:

- The Infant Toddler Checklist (ITC) can be implemented relatively quickly
- After a short implementation period and adaptation of procedures, ITC was used in all units and almost all children were screened with ITC.ITC can be an effective instrument for identifying children earlier, thus enabling timely interventions.

How this study might affect practice

- Stakeholders at the child health services should consider implementing a scientifically evaluated screening method at 18 months to enable early interventions in settings where interventions are available.

KEYWORDS:

Implementation

RE-AIM

Screening

Child Health Services

Early identification

Language disorders

Confidential: For Review Only

4

INTRODUCTION

Communication and language are the basis of human interaction and are essential for development, learning and health. Language and communication difficulties can affect a child's whole life and lead to a lower quality of life, including mental health problems, school difficulties and social exclusion.[1, 2]

Language and communication difficulties are common affecting around 10% of children in the Western world.[3] There is also considerable overlap between language and communication difficulties and other neurodevelopmental conditions.[3-5]

Early interventions for language and communication difficulties affect children's language development and are hypothesised to reduce the risk of secondary negative impacts such as disturbance in emotional and social adaptation. [6-10] To enable timely interventions, there is a need for validated and evidence-based screening instruments.[11] Several methods are available to identify children with communication and language difficulties early,[12, 13] but implementation in child health services (CHS) is still in its infancy. The Swedish CHS is regulated by a National programme[14] and reach almost all children, aiming to promote health and monitor children's development,[15] making them an excellent arena for early language screening. Children's communication and language are monitored at 18 months by asking parents if the child speaks at least 8–10 words and understands more than 8–10 words. This practice has been found to have a low sensitivity;[16] thus, more accurate screening methods are needed.

One screening instrument for identifying children with language and communication difficulties is the Communication and Symbolic Behavior Scales Developmental Profile Infant-Toddler Checklist (ITC).[17, 18] The ITC detects a range of developmental concerns, including language delay, global developmental delay and autism.[13, 19, 20] When used at the 18 month visit in Canadian primary care, the ITC had high specificity (92%) and negative predictive value (96%), and a low sensitivity (31%) and false positive rate (8%) for a developmental diagnosis at 3–5 years .[21] ITC used at the 18-month visits in a Swedish CHS showed a sensitivity of 0.85 and specificity of 0.59 which increased to 0.88 and 0.63, respectively when the ITC was combined with the nurse's assessment.[22] Further, nurses has highlighted the usability and relative advantages of using the ITC in CHS.[23]

Introducing new approaches[24] and transferring methods tested in research studies into everyday clinical practice is complex and takes time. [27] Applying an implementation framework when assessing an intervention allows one to investigate the process'*s how* and *why*, which can facilitate identifying and understanding effective

Confidential: For Review Only

components and barriers in the implementation process. [25] This, in turn, can form a rationale for future implementation.

In this study, the RE-AIM framework was applied to describe the implementation of the ITC in CHS.[26] The RE-AIM offers a structured way of describing, employing and analysing implementation processes at five key dimensions: the *reach* of the target population, *effectiveness*, *adoption*, *implementation* and *maintenance* of the implemented method.[27]

Research questions

1. What proportion of eligible children were screened with the ITC after its implementation? (Reach)
2. Did the implementation of the ITC lead to earlier referrals to Speech and language therapy (SLT) for children with suspected language or communication difficulties? Were there any sex differences in referral rates? (Effectiveness)
3. Was the ITC adopted equally across all three centres? (Adoption)
4. What actions were taken to facilitate the implementation of the ITC during the study period? (Implementation)
5. Was the implementation of the ITC sustained over time, and if so, how? (Maintenance)

METHODS

Study design

The study design used an observational study design based on medical records. As this study was based on data from medical records, no involvement of patients were possible.

Screening instrument

The ITC consists of 24 multiple-choice questions completed by the parents. It is divided in three composite scores: social, speech, and symbolic, and a total score.[17, 18] If the social or symbolic subscales or the total score are below a cut-off value of the 10th percentile (concern) and thus indicate a possible divergent communication development, the child should be referred to a specialist. If only the speech scale indicates concern, the child should be reassessed with the ITC after three months.[13] If the ITC indicates concern on the speech composite on two occasions the child should be referred.

Confidential: For Review Only

Data collection

Regional documentation regarding implementation strategies was collected from the CHS unit. Pseudonymised data, including child age at screening, sex, screening method and results, referral and age at referral to SLT and centre, were collected via the medical records system.

As the ITC-specific item-selection keywords were not applied in the medical records system during the first months after the implementation, both data based on free text entries and ITC-specific item-selections were processed. The collected ITC free-form text entries contained information on completing the ITC screening (*ITC completed*/*ITC not completed*) and potential concern for the child's communication development (*concern/no concern*). The concern data was collected through record entries, and it was not specified whether they based on the ITC *concern* item or on parent interviews during the visit. Stated reasons for not referring children with an indication of concern about failing the screening, were collected.

Research Ethics Approval

This study was conducted according to the ethical guidelines described in the Helsinki Declaration and was approved by the Swedish Ethical Review Board (*Dnr* 2021-04737). As the was based on anonymised previously collected medical record data no consent was collected.

Setting and population

The ITC 18-month screening was introduced in the Region of Gotland, the smallest county in Sweden with 500–600 children born annually. The CHS is divided into three centres in different geographical areas with different socioeconomic demographics.[28] The nurses were supported in implementation, consultation and education by a central CHS unit consisting of a nurse, a paediatrician and a psychologist.

The ITC was introduced for children born in January 2019 and onwards. Due to the summer vacation period in Sweden, three children scheduled for July visits had their 18-month visits in June 2020. However, July was considered the starting point for assessing reach and maintenance.

To answer the different research questions, two samples were used (Table 1). The first sample, answering research questions 1 and 3–5, included all children receiving their 18-month CHS visit (17–22 months) between May 2016 and April 2022 (n=2633 children, girls 48,8 %). Mean age at visit was 17.8 months (SD= 0.93). The intervention group (*n*=916, 48,7% girls), henceforth the ITC group, was exposed to ITC (June 2020–April 2022), while the control group (*n*=1717, 48,8% girls) received care as usual (May 2016–June 2020).

Confidential: For Review Only

To answer research question 2, regarding the effectiveness of the ITC in terms of early referrals (before 25 months of age), a sample was used including children who had reached at least 24 months of age or had been referred to a speech and language therapist before turning 2 months (*n*=2367). This was based on clinical experience and previous studies showing that it can take time to motivate parents to refer after screening with concern at 18 months.[23] The age limit also allowed for repeated screenings when appropriate. The control group consisted of 1717 children (48,8% girls), and the ITC group of 650 children (48.3% girls).

Table 1 Participant Characteristics

	***n* (%)**	
Full sample	Care as usual	ITC
	n = 1717	*n* = 916
Sex		
Girls	838 (48.8)	446 (48.7)
Boys	879 (51.2)	470 (51.3)
Concern		70 (7.6)
Speech		50 (5.5)
Subsample including children aged at least 24 months or referred early		
	n = 1717	*n* = 650
Sex		
Girls	838 (48.8)	314 (48.3)
Boys	879 (51.2)	336 (51.7)
Concern		58 (8.9)
Speech		35 (5.4)

Analysis

Reach was assessed through the ratio between the screened children and the total number of children in the ITC group. The effectiveness was examined through descriptive statistics with the number of children were the ITC result indicated concern, and referrals. To analyse the differences in early referral rates (effectiveness), the Chi-squared test was used. Effect size was measured through odds ratio and 95% CI, using logistic regression. Sex differences in early referral were assessed through Chi-squared test. The alpha level for the Chi-squared analyses were set to 0.05. Data were prepared and analysed using the statistical software R (version 4.2.2) and SPSS (version 28).[29, 30] Adoption rates were analysed using descriptive statistics. Implementation was assessed qualitatively through written documentation and information from staff within the central CHS unit responsible for the implementation process. Maintenance was assessed using descriptive statistics.

RESULTS

Following the structure of the implementation framework, results are presented for each domain (Table 2).

Confidential: For Review Only

Table 2. Objectives and results for each of the RE-AIM domains

	Reach	Effectiveness	Adoption	Implementation	Maintenance
**Objective**	Proportion of eligible children screened with the ITC after its implementation	Effect of the ITC implementation on earlier referrals for children with suspected language or communication difficulties	Equality of adoption across CHCs	Actions taken to facilitate the implementation of the ITC	If and how the implementation of the ITC was sustained over time
**Result**	93% screening rate (854/916 children)	Significant difference in referrals between ITC and previous method (OR = 2.07, 95% CI [1.34, 3.14]). Being a boy were associated with early referral (OR = 2.38, 95% CI [1.30, 4.36], p < 0.001)	All centres adopted the method (100% adoption rate)	• Training session<br>• Close collaboration<br>• Written procedure<br>• Automated procedure<br>• Modified medical record procedure	Time to full implementation: 3 months<br>Implementation maintained over full study period<br>Brief drop in screening rate during first 6 months

Reach

Of the 916 children in the ITC group, 854 were screened with the ITC, representing a reach of 93%. For 11 children, the ITC was reported as failing to be returned to the CHS. For the remaining 51 children, no reasons were given for the children not being screened. Please see Figure 1 for a flowchart of screening results and referrals for girls and boys.

Effectiveness

For this RE-AIM domain, the subsample was used. Of the 608 children screened with the ITC, 58 children (7.9%) had a result indicating concern. Additionally, 35 (6.3%) children had concern on the speech composite only and thus would be rescreened with the ITC three months later. However, four children were referred after the first screening. Of the remaining 31 children, 22 were not rescreened.

For 20 children screening with the ITC had been performed on two occasions. Nine of these children had concern on the speech composite only. The rescreening indicate concern for 10 children whereof 4 in the speech composite. In total 24 children with concern the ITC were not referred. For eight of these parents expressed no concern or declined the referral offer. The nurses stated no concerns regarding the child's communication development for six. Of the remaining nine children no reasons were given. Additionally, 22 children were not referred or only screened with ITC once after a positive screening.

Analysing the subsample of children aged at least 24 months or referred early, a significant relationship between screening method and early referral was detected, where the ITC was associated with more early referrals

Confidential: For Review Only

($\chi 2$ [n=2,367] =93.2, p<0.001). Referral rates were 0.4% in the care as usual group and 6.9% in the ITC group. The odds for early referral were substantially higher for children screened with the ITC (OR = 18.17, 95% CI [8.15, 40.51], $p < 0.001$). Further, being a boy was associated with an earlier referral (OR = 2.38, 95% CI [1.30, 4.36], $p < 0.001$). In the control group, 28.6% of the referred children were girls, while this rate was 28.9% in the ITC group. These rates did not significantly differ from each other.

Adoption

All centres implemented ITC, resulting in a 100% adoption rate at setting level, which remained unchanged during the study period. At the centre level, there was variation in the proportion of children screened (range 92–97%) with the highest adherence in the most socio-economically challenged area.

Implementation and implementation strategies

The implementation was performed in collaboration between the central CHS unit and the SLT services. Prior to the implementation of the ITC, staff attended training sessions and workshops on parent-child relationships, early communication development, the importance of early intervention and how to perform the ITC. A written procedure was developed, including referrals and re-screenings. Shortly after implementing the ITC, a medical record system was modified to include an ITC-specific template with fixed response options for record entry. Before the modification, the nurses entered the results of the ITC screening in free-form text fields into the medical record. Further, the medical record system automatically included the ITC with the invitation letter to the 18-month visit.

Maintenance

During the study, the CHS unit, mainly through a designated project manager, kept close contact with the nurses, offering support feedback and monitoring the implementation process. The screening procedure instructions were continuously updated and improved throughout the implementation. The use of the ITC was sustained over time during the entire study period and is, as of 2023, still part of routine practice across the county.

The consistency of implementation varied in the early phase of implementation, as the proportion of children screened was initially low: in the first three months, 20% of the children were not screened. Thereafter, the rate of screened children varied between 94% and 100% until January 2022 (Figure 2). During this month, the rate of screened children dropped to 87%. This coincided with the start of a short-term employment of a substitute nurse. By March 2022, the rate of screened children had increased to 94%.

Confidential: For Review Only

DISCUSSION

Introducing new procedures and methods to everyday clinical practice is often complicated and challenging.[31] It is, therefore, essential to monitor the implementation processes. This study aimed to enhance knowledge regarding implementing the ITC through the RE-AIM framework. [26, 27] A vast majority of the children were screened with the ITC after its introduction (93%). More children were referred early to speech and language assessment after the introduction of the ITC compared with care as usual. The overall adoption rate was 100%, with all three centres adopting the method from its introduction. Strategies for successful implementation included a training session, close collaboration with external parties, written and automated procedures, and modified procedures regarding medical record keeping. The implementation maintenance was sustained over time.

The reach of the ITC was high; 93% of children who visited the CHS since the introduction were screened with the ITC. One risk of using screening instruments is that children are not identified equally across demographics.[32] No individual socio-demographic data were available in this study. Therefore, it is impossible to conclude whether children are screened and identified equally. However, the three centres were located in areas with diverse socioeconomic makeup, and results suggested that adoption was highest in the most socio-economically challenged areas.[28]

There was a large difference in referrals between girls and boys with an OR at 2.38, using both usual care and the ITC, although the difference tended to be smaller after the introduction of ITC. Language and communication difficulties have been shown to be more common in boys than in girls[33] and a recent study found an adjusted OR at 2.15 for boys using ITC.[34] Other studies have found that the sex differences are often minor and non-significant.[3] On the other hand, the boy-girl ratio for receiving intervention is significant,[35] which can result from differences in identification. It is essential to study reasons for sex differences in identification and referral in future studies.

Implementation took place in inter-organisational and inter-professional collaboration. This presumably positively affected the implementation and facilitated planning and communication in the short and long term. As anticipated, the implementation had a start-up period of three months before almost all children were screened with the ITC, a period considered short compared with other implementations.[36] Preparations for the implementation included workshops and training for the nurses. This can be seen as a beneficial factor to the

Confidential: For Review Only

high adoption rate and reach and an important part of the implementation, as the nurses were relatively well prepared for the implementation process.

The central CHS unit included a designated project manager, who followed the implementation of the ITC closely. The continuous improvement of the written procedures and routines may have facilitated the implementation, created reassurance for the nurses and reduced the risk of incorrect practice. The automated printing and including the ITC with the invitation letter to the parents reduced the risk of failing to send out the questionnaires. Further, the revised medical journal template reminded the nurses and facilitated correct record-keeping.

The decrease in the proportion of screened children during staff turnover may indicate the importance of ongoing training and supervision to facilitate maintenance of screening. However, since the number of children having health visits each month was relatively low, one needs to exercise caution when interpreting Figure 2.

Not all children who should have been rescreened after concern on the speech composite were rescreened. and some children who had a ITC result indicating concern were not referred to SLT. While this was mainly explained through parents declining referral or the nurses' clinical assessment for some children, no reason was given for not sending a referral for nine of the children. Adherence to referral guidelines can be low and might depend on family demographics.[37] Even though ITC has been described as giving parents insight into their child's development and facilitated referrals, it can be difficult to motivate parents referral to SLT at 18 months.[23] Professionals sometimes hold off on referral, even when parents express concern.[38] The psychometric properties of the ITC in the Swedish setting with a somewhat low specificity could be a possible contributing factor to the lower referral rate.[22] Other studies have shown a high specificity and low sensitivity of ITC screening at 18 months.[21] However, methodological differences may account for the discrepancies to some extent. Problems with specificity and sensitivity highlight the need for repeated screening and health surveillance. Screening may need to be supplemented with the nurse's clinical observation and a dialogue with the parent.

The goal of implementing the ITC was to ensure that children needing support in their communication and language development would be identified earlier and referred to specialist services such as SLT. For early referrals to be meaningful, interventions must be available in conjunction with the referrals. In the Region of Gotland, such a timely intervention is available, which is not the case in all of Sweden, where the waiting times for SLT interventions can be years long.[39]

Confidential: For Review Only

12

One basic principle of screening is that only children needing interventions should be identified,[40] which is especially important when resources are scarce. This puts extra demands on the use of clinically valid screening instruments. The ITC could be one such instrument, as the analyses show that the referral rate was equal before and after the implementation of the ITC but that the referrals occured when the children were younger. Future studies should assess how ITC affects the care process for children how the ITC relates to later language screenings.

CONCLUSIONS

The implementation of the ITC was characterised by a high reach, higher rates of early referrals, complete adoption, and sustained maintenance over time. Thus, the ITC implementation process was successful in relation to the RE-AIM domains. The successful implementation was facilitated through training, improvements in the medical record system, close monitoring of the implementation and written procedures that may have contributed to the outcome.

The ITC can be used for early detection of language and communication difficulties and hence help families receive timely interventions. Depending on context, a similar implementation could be beneficial, as there is a general need for earlier screening of language and communication difficulties to enable timely interventions.

CONTRIBUTORS

AF, AD, AL: conceptualised, designed the study and drafted the original manuscript. AF and AD: data analysis, AL: performed electronic health record data extraction. All authors reviewed and approved the final manuscript as submitted. AF willingly accepted the role of guarantor for this study.

FUNDING INFORMATION

The study was funded by Länsförsäkringar Gotland

ACKNOWLEDGEMENTS

The authors would like to thank the child health service in the Region of Gotland for their contribution.

FIGURE LEGENDS

Figure 1 Screening results and referrals for girls and boys

Figure 2 Screening rates across implementation time

Confidential: For Review Only

13

REFERENCES

1.McCormack, et al., *A systematic review of the association between childhood speech impairment and participation across the lifespan.* Int. J. Speech Lang. Pathol., 2009.
2.den Bedem, et al., *Depressive symptoms and emotion regulation strategies in children with and without developmental language disorder: a longitudinal study.* Int. J. Lang. Commun. Disord., 2018.
3.Norbury, et al., *The impact of nonverbal ability on prevalence and clinical presentation of language disorder: evidence from a population study.* J. Child Psychol, 2016.
4.Sciberras, et al., *Language problems in children with ADHD: A community-based study.* Pediatrics (Evanston), 2014.
5.McGrath, et al., *Children with Comorbid Speech Sound Disorder and Specific Language Impairment are at Increased Risk for Attention-Deficit/Hyperactivity Disorder.* J. Abnorm. Child Psychol., 2007.
6.Warren, et al., *A Systematic Review of Early Intensive Intervention for Autism Spectrum Disorders.* Pediatrics, 2011.
7.Roberts, et al., *Association of Parent Training With Child Language Development A Systematic Review and Meta-analysis.* JAMA PEDIATRICS, 2019.
8.Dubin and Lieberman-Betz, *Naturalistic Interventions to Improve Prelinguistic Communication for Children with Autism Spectrum Disorder: a Systematic Review.* Review Journal of Autism and Developmental Disorders, 2020.
9.Novak and Morgan, *Chapter 23 - High-risk follow-up: Early intervention and rehabilitation*, in *Handb. Clin. Neurol.*, de Vries and Glass, Editors. 2019, Elsevier.
10.Rinaldi, et al., *Efficacy of the Treatment of Developmental Language Disorder: A Systematic Review.* Brain Sci., 2021.
11.Horiuchi, et al., *"Caregivers' awareness matters": Call for a child-centered approach to early detection of autism spectrum disorder through well-child visits in Japan.* Child Care Health Dev, 2023.
12.Sim, et al., *Predictive validity of preschool screening tools for language and behavioural difficulties: A PRISMA systematic review.* PLoS One, 2019.
13.Wetherby, et al., *Validation of the Infant—Toddler Checklist as a broadband screener for autism spectrum disorders from 9 to 24 months of age.* Autism, 2008.
14.Tell. *Swedish Child health services.*
15.Wallby and Hjern, *Child health care uptake among low-income and immigrant families in a Swedish county.* Acta Paediatr., 2011.
16.Westerlund, et al., *Can Severely Language Delayed 3-Year-Olds Be Identified at 18 Months? Evaluation of a Screening Version of the MacArthur-Bates Communicative Development Inventories.* J. Speech. Lang. Hear. Res., 2006.
17.Wetherby and Prizant, *Communication and Symbolic Behavior Scales Developmental Profile, First Normed Edition*. 2002,
18.Wetherby and Prizant, *Communication and Symbolic Behavior Scales Developmental Profile Infant/Toddler Checklist.* 2001.
19.Wetherby, et al., *Validity and reliability of the communication and symbolic behavior scales developmental profile with very young children.* J. Speech. Lang. Hear. Res., 2002.
20.Wetherby, et al., *Early identification of children with communication disorders: concurrent and predictive validity of the CSBS Developmental Profile.* Infants & Young Children: An Interdisciplinary Journal of Early Childhood Intervention, 2003.

Confidential: For Review Only

21. Borkhoff, et al., *Predictive validity of the Infant Toddler Checklist in primary care at the 18-month visit and developmental diagnosis at 3–5 years: a prospective cohort study.* BMJ Paediatrics Open, 2022.
22. Fäldt, et al., *Infant-Toddler Checklist identifies 18-month-old children with communication difficulties in the Swedish child healthcare setting.* Acta Paediatr., 2021.
23. Fäldt, et al., *Nurses' experiences of screening for communication difficulties at 18 months of age.* Acta Paediatr., 2019.
24. Woolf, *The meaning of translational research and why it matters.* Jama, 2008.
25. Rubenstein and Pugh, *Strategies for promoting organizational and practice change by advancing implementation research.* Journal of General Internal Medicine, 2006.
26. Glasgow, et al., *Evaluating the public health impact of health promotion interventions: the RE-AIM framework.* American journal of public health (1971), 1999.
27. Glasgow, et al., *RE-AIM Planning and Evaluation Framework: Adapting to New Science and Practice With a 20-Year Review.* Frontiers in Public Health, 2019.
28. DEMOS. *Segregationsbarometern [Segregation barometer].*
29. R Core Team, *R: A language and environment for statistical computing*. 2022, R Foundation for Statistical Computing: Vienna, Austria.
30. IBM Corp., *IBM SPSS Statistics for Macintosh*. 2022: Armonk, NY.
31. Damschroder, et al., *Fostering implementation of health services research findings into practice: a consolidated framework for advancing implementation science.* Implementation science : IS, 2009.
32. Guthrie, et al., *Accuracy of Autism Screening in a Large Pediatric Network.* Pediatrics, 2019.
33. Rudolph, *Case History Risk Factors for Specific Language Impairment: A Systematic Review and Meta-Analysis.* Am. J. Speech Lang. Pathol., 2017.
34. Nurse, et al., *Association Between Family Income and Positive Developmental Screening Using the Infant Toddler Checklist at the 18-Month Health Supervision Visit.* The Journal of Pediatrics, 2024.
35. Morgan, et al., *Cross-Cohort Evidence of Disparities in Service Receipt for Speech or Language Impairments.* Except. Child., 2017.
36. Morris, et al., *The answer is 17 years, what is the question: understanding time lags in translational research.* J. R. Soc. Med., 2011.
37. Wallis, et al., *Adherence to screening and referral guidelines for autism spectrum disorder in toddlers in pediatric primary care.* PLoS One, 2020.
38. Weitlauf, et al., *Black Families' Experiences of Developmental Screening: Review of Well-Child Visits to Inform Enhanced Autism Spectrum Disorder Risk Assessment.* J. Dev. Behav. Pediatr., 2022.
39. Marjomaa, *Lång kö till logoped för barn i Västra Götaland [Long waiting time to get SLT service for children in Västra Götaland]*, in *SVT Väst*. 2021: Göteborg.
40. Wilson and Jungner, *Principles and practice of screening for disease.* 1968, WHO: Geneva.

Confidential: For Review Only

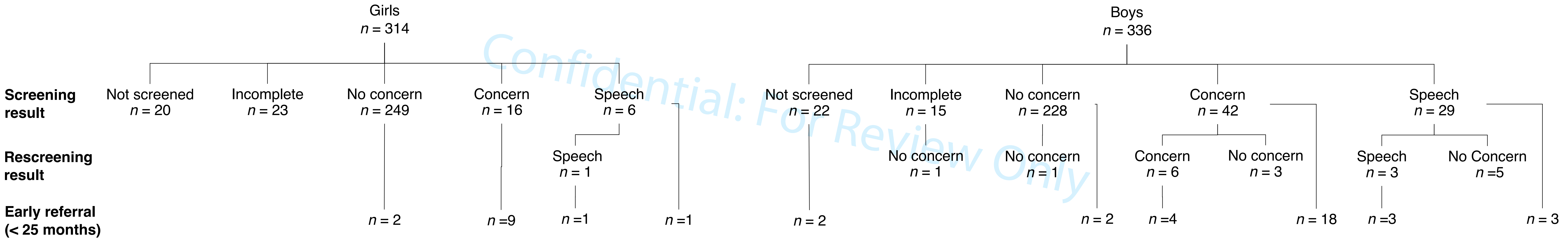

Confidential: For Review Only

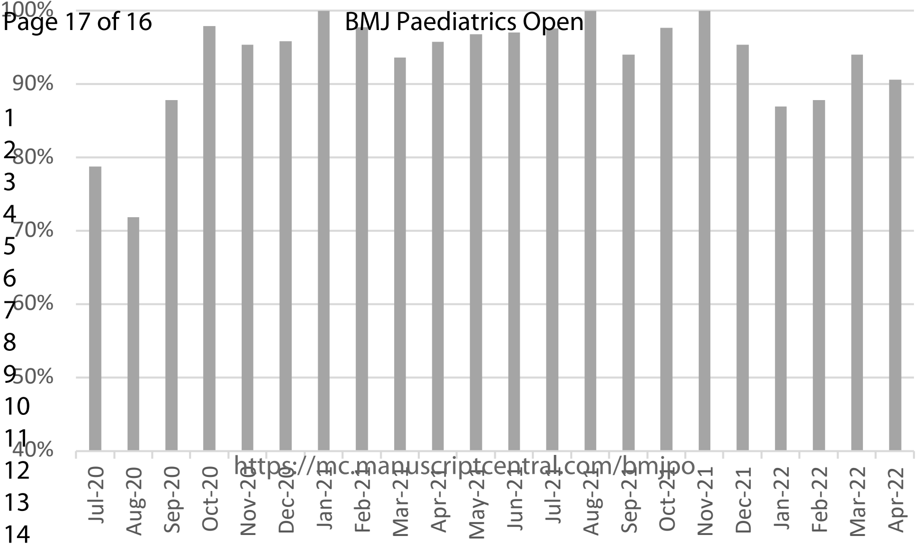

https://mc.manuscriptcentral.com/bmjpo

BMJ Paediatrics Open

Implementation of the Infant-Toddler Checklist in Swedish child health services at 18 months: an observational study

Journal:	*BMJ Paediatrics Open*
Manuscript ID	bmjpo-2023-002406.R2
Article Type:	Original research
Date Submitted by the Author:	07-Mar-2024
Complete List of Authors:	Dahlberg, Anton; Uppsala University, Child Health and Parenting (CHAP), Department of Public Health and Caring Sciences<br>Levin, Anna; Uppsala University, Child Health and Parenting (CHAP), Department of Public Health and Caring Sciences<br>Fäldt, Anna; Uppsala Universitet, Dept of Public Health and Caring Sciences
Keywords:	Screening, Infant

SCHOLARONE™
Manuscripts

I, the Submitting Author has the right to grant and does grant on behalf of all authors of the Work (as defined in the below author licence), an exclusive licence and/or a non-exclusive licence for contributions from authors who are: i) UK Crown employees; ii) where BMJ has agreed a CC-BY licence shall apply, and/or iii) in accordance with the terms applicable for US Federal Government officers or employees acting as part of their official duties; on a worldwide, perpetual, irrevocable, royalty-free basis to BMJ Publishing Group Ltd ("BMJ") its licensees and where the relevant Journal is co-owned by BMJ to the co-owners of the Journal, to publish the Work in this journal and any other BMJ products and to exploit all rights, as set out in our licence.

The Submitting Author accepts and understands that any supply made under these terms is made by BMJ to the Submitting Author unless you are acting as an employee on behalf of your employer or a postgraduate student of an affiliated institution which is paying any applicable article publishing charge ("APC") for Open Access articles. Where the Submitting Author wishes to make the Work available on an Open Access basis (and intends to pay the relevant APC), the terms of reuse of such Open Access shall be governed by a Creative Commons licence – details of these licences and which Creative Commons licence will apply to this Work are set out in our licence referred to above.

Other than as permitted in any relevant BMJ Author's Self Archiving Policies, I confirm this Work has not been accepted for publication elsewhere, is not being considered for publication elsewhere and does not duplicate material already published. I confirm all authors consent to publication of this Work and authorise the granting of this licence.

Confidential: For Review Only

Implementation of the Infant-Toddler Checklist in Swedish child health services at 18 months: an observational study

Anton DAHLBERG[a], Anna LEVIN[a], Anna Erica FÄLDT[a]*

*Corresponding author

[a]Child Health and Parenting (CHAP), Department of Public Health and Caring Sciences, Uppsala University, Box 564, 751 22 UPPSALA Sweden

Anna Fäldt

anna.faldt@uu.se Telephone: +46 76 776 77 55

orcid: 0000-0001-7257-8758

Anton Dahlberg

Anton.dahlberg@uu.se Telephone: +46 18-471 6574

orcid:0000-0003-0136-8862

Anna Levin

Anna.levin@gotland.se Telephone: +46 498-26 85 01

Competing interest declaration:

Author 2, Anna Levin, was involved in the planning and implementing of the ITC in the region of Gotland.

Confidential: For Review Only

ABSTRACT

Background: Communication and language disorders are common conditions that emerge early and negatively impact quality of life across the life course. Early identification may be facilitated using a validated screening tool such as the Infant Toddler Checklist (ITC). We introduced the ITC at the 18-month visit at child health services in a Swedish county. Using the RE-AIM implementation framework, this study assessed the implementation of the ITC according to five key dimensions: reach, effectiveness, adoption, implementation, and maintenance.

Methods: This observational study used medical records at child health services as data source. Data were collected from children who visited a child health nurse at 17–22 months. The sample included 2633 children with a mean age of 17.8 months, 1717 in the pre-implementation group and 916 in the post-implementation group. We calculated ITC completion rate (reach) and use at each site (adoption). We compared rates of referral to speech and language therapy (effectiveness) before and after implementation of the ITC using Odds Ratio (OR) and 95% Confidence Intervals (CI). We described actions to facilitate implementation and maintenance of ITC screening over time.

Results: The overall screening rate was 93% (reach) which increased from 80% initially to 94% at the end of the 2-year period (maintenance). All centres used the ITC (adoption). The ITC screen positive rate was 14%. Of children who had reached at least 24 months (n=2367), referral rate was 0.4% pre-implementation versus 6.9% post-implementation (OR = 18.17, 95% CI [8.15, 40.51], $p < 0.001$) (effectiveness). Implementation strategies included training sessions, collaboration, written and automatic procedures and modifications to the medical records system.

Conclusion: The implementation of the ITC was associated with high reach, higher referral rate, complete adoption, and sustained maintenance over time.

KEY MESSAGE

What is known about the subject:

- Early language assessment is essential for offering timely interventions to children with communication disorders.
- Implementing and maintaining new screening procedures in healthcare is difficult and research on this is scarce.

Confidential: For Review Only

3

What this study adds:

- The Infant Toddler Checklist (ITC) can be implemented relatively quickly
- After a short implementation period and adaptation of procedures, ITC was used in all units and almost all children were screened with ITC.ITC can be an effective instrument for identifying children earlier, thus enabling timely interventions.

How this study might affect practice

- Stakeholders at the child health services should consider implementing a scientifically evaluated screening method at 18 months to enable early interventions in settings where interventions are available.

KEYWORDS:

Implementation

RE-AIM

Screening

Child Health Services

Early identification

Language disorders

Confidential: For Review Only

INTRODUCTION

Communication and language are the basis of human interaction and are essential for development, learning and health. Language and communication difficulties can affect a child's whole life and lead to a lower quality of life, including mental health problems, school difficulties and social exclusion.[1, 2]

Language and communication difficulties are common affecting around 10% of children in the Western world.[3] There is also considerable overlap between language and communication difficulties and other neurodevelopmental conditions.[3-5]

Early interventions for language and communication difficulties affect children's language development and are hypothesised to reduce the risk of secondary negative impacts such as disturbance in emotional and social adaptation. [6-10] To enable timely interventions, there is a need for validated and evidence-based screening instruments.[11] Several methods are available to identify children with communication and language difficulties early,[12, 13] but implementation in child health services (CHS) is still in its infancy. The Swedish CHS is regulated by a National programme[14] and reaches almost all children, aiming to promote health and monitor children's development,[15] making them an excellent arena for early language screening. Children's communication and language are monitored at 18 months by asking parents if the child speaks at least 8–10 words and understands more than 8–10 words. This practice has been found to have a low sensitivity;[16] thus, more accurate screening methods are needed.

One screening instrument for identifying children with language and communication difficulties is the Communication and Symbolic Behavior Scales Developmental Profile Infant-Toddler Checklist (ITC).[17, 18] The ITC detects a range of developmental concerns, including language delay, global developmental delay and autism.[13, 19, 20] When used at the 18 month visit in Canadian primary care, the ITC had high specificity (92%) and negative predictive value (96%), and a low sensitivity (31%) and false positive rate (8%) for a developmental diagnosis at 3–5 years .[21] ITC used at the 18-month visits in a Swedish CHS showed a sensitivity of 0.85 and specificity of 0.59 which increased to 0.88 and 0.63, respectively when the ITC was combined with the nurse’s assessment.[22] Further, nurses have highlighted the usability and relative advantages of using the ITC in CHS.[23]

Introducing new approaches[24] and transferring methods tested in research studies into everyday clinical practice is complex and takes time. [27] Applying an implementation framework when assessing an intervention allows one to investigate the process’*s how* and *why*, which can facilitate identifying and understanding effective

Confidential: For Review Only

components and barriers in the implementation process. [25] This, in turn, can form a rationale for future implementation.

In this study, the RE-AIM framework was applied to describe the implementation of the ITC in CHS.[26] The RE-AIM offers a structured way of describing, employing and analysing implementation processes at five key dimensions: the *reach* of the target population, *effectiveness*, *adoption*, *implementation* and *maintenance* of the implemented method.[27]

Research questions

1. What proportion of eligible children were screened with the ITC after its implementation? (Reach)
2. Did the implementation of the ITC lead to earlier referrals to Speech and language therapy (SLT) for children with suspected language or communication difficulties? Were there any sex differences in referral rates? (Effectiveness)
3. Was the ITC adopted equally across all three centres? (Adoption)
4. What actions were taken to facilitate the implementation of the ITC during the study period? (Implementation)
5. Was the implementation of the ITC sustained over time, and if so, how? (Maintenance)

METHODS

Study design

The study design used an observational study design based on medical records. As this study was based on data from medical records, involvement of patients was not possible.

Screening instrument

The ITC consists of 24 multiple-choice questions completed by the parents. It is divided in three composite scores: social, speech, and symbolic, and a total score.[17, 18] If the social or symbolic subscales or the total score are below a cut-off value of the 10th percentile (concern) and thus indicate a possible divergent communication development, the child should be referred to a specialist. If only the speech scale indicates concern, the child should be reassessed with the ITC after three months.[13] If the ITC indicates concern on the speech composite on two occasions the child should be referred.

Confidential: For Review Only

6

Data collection

Regional documentation regarding implementation strategies was collected from the CHS unit. Pseudonymised data, including child age at screening, sex, screening method and results, referral and age at referral to SLT and centre, were collected via the medical records system.

As the ITC-specific item-selection keywords were not applied in the medical records system during the first months after the implementation, both data based on free text entries and ITC-specific item-selections were processed. The collected ITC free-form text entries contained information on completing the ITC screening (*ITC completed*/*ITC not completed*) and potential concern for the child's communication development (*concern/no concern*). The concern data was collected through record entries, and it was not specified whether they based on the ITC *concern* item or on parent interviews during the visit. Stated reasons for not referring children with an indication of concern about failing the screening, were collected.

Research Ethics Approval

This study was conducted according to the ethical guidelines described in the Helsinki Declaration and was approved by the Swedish Ethical Review Board (*Dnr* 2021-04737). As the study was based on anonymised previously collected medical record data no consent was collected.

Setting and population

The ITC 18-month screening was introduced in the Region of Gotland, the smallest county in Sweden with 500–600 children born annually. The CHS is divided into three centres in different geographical areas with different socioeconomic demographics.[28] The nurses were supported in implementation, consultation and education by a central CHS unit consisting of a nurse, a paediatrician and a psychologist.

The ITC was introduced for children born in January 2019 and onwards. Due to the summer vacation period in Sweden, three children scheduled for July visits had their 18-month visits in June 2020. However, July was considered the starting point for assessing reach and maintenance.

To answer the different research questions, two samples were used (Table 1). The first sample, answering research questions 1 and 3–5, included all children receiving their 18-month CHS visit (17–22 months) between May 2016 and April 2022 (n=2633 children, girls 48.8 %). Mean age at visit was 17.8 months (SD= 0.93). The intervention group (*n*=916, 48.7% girls), henceforth the ITC group, was exposed to ITC (June 2020–April 2022), while the control group (*n*=1717, 48.8% girls) received care as usual (May 2016–June 2020).

Confidential: For Review Only

149 To answer research question 2, regarding the effectiveness of the ITC in terms of early referrals (before 25 months of age), a sample was used including children who had reached at least 24 months of age or had been referred to a speech and language therapist before turning 24 months (*n*=2367). This was based on clinical experience and previous studies showing that it can take time to motivate parents to refer after screening with concern at 18 months.[23] The age limit also allowed for repeated screenings when appropriate. The control group consisted of 1717 children (48.8% girls), and the ITC group of 650 children (48.3% girls).

Table.1 Characteristics of the participants in the two samples

	***n* (%)**	
Full sample	Care as usual	ITC
	n = 1717	*n* = 916
Sex		
Girls	838 (48.8)	446 (48.7)
Boys	879 (51.2)	470 (51.3)
Concern		70 (7.6)
Speech		50 (5.5)
Subsample including children aged at least 24 months or referred early		
	n = 1717	*n* = 650
Sex		
Girls	838 (48.8)	314 (48.3)
Boys	879 (51.2)	336 (51.7)
Concern		58 (8.9)
Speech		35 (5.4)

155

156 **Analysis**

157 Reach was assessed through the ratio between the screened children and the total number of children in the ITC group. The effectiveness was examined through descriptive statistics with the number of children where the ITC result indicated concern, and referrals. To analyse the differences in early referral rates (effectiveness), the Chi-squared test was used. Effect size was measured through odds ratio and 95% CI, using logistic regression. Sex differences in early referral were assessed through Chi-squared test. The alpha level for the Chi-squared analyses were set to 0.05. Data were prepared and analysed using the statistical software R (version 4.2.2) and SPSS (version 28).[29, 30] Adoption rates were analysed using descriptive statistics. Implementation was assessed qualitatively through written documentation and information from staff within the central CHS unit responsible for the implementation process. Maintenance was assessed using descriptive statistics.

166 **RESULTS**

167 Following the structure of the implementation framework, results are presented for each domain (Table 2).

Confidential: For Review Only

8

Table 2. Objectives and results for each of the RE-AIM domains

	Reach	Effectiveness	Adoption	Implementation	Maintenance
Objective	Proportion of eligible children screened with the ITC after its implementation	Effect of the ITC implementation on earlier referrals for children with suspected language or communication difficulties	Equality of adoption across CHCs	Actions taken to facilitate the implementation of the ITC	If and how the implementation of the ITC was sustained over time
Result	93% screening rate (854/916 children)	Significant difference in referrals between ITC and previous method (OR = 2.07, 95% CI [1.34, 3.14]). Being a boy were associated with early referral (OR = 2.38, 95% CI [1.30, 4.36], p < 0.001)	All centres adopted the method (100% adoption rate)	• Training session<br>• Close collaboration<br>• Written procedure<br>• Automated procedure<br>• Modified medical record procedure	Time to full implementation: 3 months<br>Implementation maintained over full study period<br>Brief drop in screening rate during first 6 months

168

169 **Reach**

170 Of the 916 children in the ITC group, 854 were screened with the ITC, representing a reach of 93%. For 11
171 children, the ITC was reported as failing to be returned to the CHS. For the remaining 51 children, no reasons
172 were given for the children not being screened. Please see Figure 1 for a flowchart of screening results and
173 referrals for girls and boys.

174 **Effectiveness**

175 For this RE-AIM domain, the subsample was used. Of the 608 children screened with the ITC, 58 children
176 (7.9%) had a result indicating concern. Additionally, 35 (6.3%) children had concern on the speech composite
177 only and thus would be rescreened with the ITC three months later. However, four children were referred after
178 the first screening. Of the remaining 31 children, 22 were not rescreened.

179 For 20 children screening with the ITC had been performed on two occasions. Nine of these children had
180 concern on the speech composite only. The rescreening indicate concern for 10 children whereof 4 in the speech
181 composite. In total 24 children with concern the ITC were not referred. For eight of these parents expressed no
182 concern or declined the referral offer. The nurses stated no concerns regarding the child's communication
183 development for six. Of the remaining nine children no reasons were given. Additionally, 22 children were not
184 referred or only screened with ITC once after a positive screening.

185 Analysing the subsample of children aged at least 24 months or referred early, a significant relationship between
186 screening method and early referral was detected, where the ITC was associated with more early referrals

Confidential: For Review Only

($\chi2$ [n=2,367] =93.2, p<0.001). Referral rates were 0.4% in the care as usual group and 6.9% in the ITC group. The odds for early referral were substantially higher for children screened with the ITC (OR = 18.17, 95% CI [8.15, 40.51], $p < 0.001$). Further, being a boy was associated with an earlier referral (OR = 2.38, 95% CI [1.30, 4.36], $p < 0.001$). In the control group, 28.6% of the referred children were girls, while this rate was 28.9% in the ITC group. These rates did not significantly differ from each other.

Adoption

All centres implemented ITC, resulting in a 100% adoption rate at setting level, which remained unchanged during the study period. At the centre level, there was variation in the proportion of children screened (range 92–97%) with the highest adherence in the most socio-economically challenged area.

Implementation and implementation strategies

The implementation was performed in collaboration between the central CHS unit and the SLT services. Prior to the implementation of the ITC, staff attended training sessions and workshops on parent-child relationships, early communication development, the importance of early intervention and how to perform the ITC. A written procedure was developed, including referrals and re-screenings. Shortly after implementing the ITC, a medical record system was modified to include an ITC-specific template with fixed response options for record entry. Before the modification, the nurses entered the results of the ITC screening in free-form text fields into the medical record. Further, the medical record system automatically included the ITC with the invitation letter to the 18-month visit.

Maintenance

During the study, the CHS unit, mainly through a designated project manager, kept close contact with the nurses, offering support feedback and monitoring the implementation process. The screening procedure instructions were continuously updated and improved throughout the implementation. The use of the ITC was sustained over time during the entire study period and is, as of 2023, still part of routine practice across the county.

The consistency of implementation varied in the early phase of implementation, as the proportion of children screened was initially low: in the first three months, 20% of the children were not screened. Thereafter, the rate of screened children varied between 94% and 100% until January 2022 (Figure 2). During this month, the rate of screened children dropped to 87%. This coincided with the start of a short-term employment of a substitute nurse. By March 2022, the rate of screened children had increased to 94%.

Confidential: For Review Only

DISCUSSION

Introducing new procedures and methods to everyday clinical practice is often complicated and challenging.[31] It is, therefore, essential to monitor the implementation processes. This study aimed to enhance knowledge regarding implementing the ITC through the RE-AIM framework. [26, 27] A vast majority of the children were screened with the ITC after its introduction (93%). More children were referred early to speech and language assessment after the introduction of the ITC compared with care as usual. The overall adoption rate was 100%, with all three centres adopting the method from its introduction. Strategies for successful implementation included a training session, close collaboration with external parties, written and automated procedures, and modified procedures regarding medical record keeping. The implementation maintenance was sustained over time.

The reach of the ITC was high; 93% of children who visited the CHS since the introduction were screened with the ITC. One risk of using screening instruments is that children are not identified equally across demographics.[32] No individual socio-demographic data were available in this study. Therefore, it is impossible to conclude whether children are screened and identified equally. However, the three centres were located in areas with diverse socioeconomic makeup, and results suggested that adoption was highest in the most socio-economically challenged areas.[28]

There was a large difference in referrals between girls and boys with an OR at 2.38, using both usual care and the ITC, although the difference tended to be smaller after the introduction of ITC. Language and communication difficulties have been shown to be more common in boys than in girls[33] and a recent study found an adjusted OR at 2.15 for boys using ITC.[34] Other studies have found that the sex differences are often minor and non-significant.[3] On the other hand, the boy-girl ratio for receiving intervention is significant,[35] which can result from differences in identification. It is essential to study reasons for sex differences in identification and referral in future studies.

Implementation took place with inter-organisational and inter-professional collaboration. This presumably positively affected the implementation and facilitated planning and communication in the short and long term. As anticipated, the implementation had a start-up period of three months before almost all children were screened with the ITC, a period considered short compared with other implementations.[36] Preparations for the implementation included workshops and training for the nurses. This can be seen as a beneficial factor to

Confidential: For Review Only

the high adoption rate and reach and an important part of the implementation, as the nurses were relatively well prepared for the implementation process.

The central CHS unit included a designated project manager, who followed the implementation of the ITC closely. The continuous improvement of the written procedures and routines may have facilitated the implementation, created reassurance for the nurses and reduced the risk of incorrect practice. The automated printing and including the ITC with the invitation letter to the parents reduced the risk of failing to send out the questionnaires. Further, the revised medical journal template reminded the nurses and facilitated correct record-keeping.

The decrease in the proportion of screened children during staff turnover may indicate the importance of ongoing training and supervision to facilitate maintenance of screening. However, since the number of children having health visits each month was relatively low, one needs to exercise caution when interpreting Figure 2.

Not all children who should have been rescreened after concern on the speech composite were rescreened. and some children who had a ITC result indicating concern were not referred to SLT. While this was mainly explained through parents declining referral or the nurses' clinical assessment for some children, no reason was given for not sending a referral for nine of the children. Adherence to referral guidelines can be low and might depend on family demographics.[37] Even though ITC has been described as giving parents insight into their child's development and facilitated referrals, it can be difficult to motivate parents referral to SLT at 18 months.[23] Professionals sometimes hold off on referral, even when parents express concern.[38] The psychometric properties of the ITC in the Swedish setting with a somewhat low specificity could be a possible contributing factor to the lower referral rate.[22] Other studies have shown a high specificity and low sensitivity of ITC screening at 18 months.[21] However, methodological differences may account for the discrepancies to some extent. Problems with specificity and sensitivity highlight the need for repeated screening and health surveillance. Screening may need to be supplemented with the nurse's clinical observation and a dialogue with the parent.

The goal of implementing the ITC was to ensure that children needing support in their communication and language development would be identified earlier and referred to specialist services such as SLT. For early referrals to be meaningful, interventions must be available in conjunction with the referrals. In the Region of Gotland, such a timely intervention is available, which is not the case in all of Sweden, where the waiting times for SLT interventions can be years long.[39]

Confidential: For Review Only

One basic principle of screening is that only children needing interventions should be identified,[40] which is especially important when resources are scarce. This puts extra demands on the use of clinically valid screening instruments. The ITC could be one such instrument, as the analyses show that the referral rate was equal before and after the implementation of the ITC but that the referrals occured when the children were younger. Future studies should assess how ITC affects the care process for children how the ITC relates to later language screenings.

CONCLUSIONS

The implementation of the ITC was characterised by a high reach, higher rates of early referrals, complete adoption, and sustained maintenance over time. Thus, the ITC implementation process was successful in relation to the RE-AIM domains. The successful implementation was facilitated through training, improvements in the medical record system, close monitoring of the implementation and written procedures that may have contributed to the outcome.

The ITC can be used for early detection of language and communication difficulties and hence help families receive timely interventions. Depending on context, a similar implementation could be beneficial, as there is a general need for earlier screening of language and communication difficulties to enable timely interventions.

CONTRIBUTORS

AF, AD, AL: conceptualised, designed the study and drafted the original manuscript. AF and AD: data analysis, AL: performed electronic health record data extraction. All authors reviewed and approved the final manuscript as submitted. AF willingly accepted the role of guarantor for this study.

FUNDING INFORMATION

The study was funded by Länsförsäkringar Gotland

ACKNOWLEDGEMENTS

The authors would like to thank the child health service in the Region of Gotland for their contribution.

FIGURE LEGENDS

Figure 1 Flowchart of screening results and referrals for girls and boys

Figure 2 Screening rates across implementation time

Confidential: For Review Only

13

REFERENCES

1.McCormack, et al., *A systematic review of the association between childhood speech impairment and participation across the lifespan.* Int. J. Speech Lang. Pathol., 2009.
2.den Bedem, et al., *Depressive symptoms and emotion regulation strategies in children with and without developmental language disorder: a longitudinal study.* Int. J. Lang. Commun. Disord., 2018.
3.Norbury, et al., *The impact of nonverbal ability on prevalence and clinical presentation of language disorder: evidence from a population study.* J. Child Psychol, 2016.
4.Sciberras, et al., *Language problems in children with ADHD: A community-based study.* Pediatrics (Evanston), 2014.
5.McGrath, et al., *Children with Comorbid Speech Sound Disorder and Specific Language Impairment are at Increased Risk for Attention-Deficit/Hyperactivity Disorder.* J. Abnorm. Child Psychol., 2007.
6.Warren, et al., *A Systematic Review of Early Intensive Intervention for Autism Spectrum Disorders.* Pediatrics, 2011.
7.Roberts, et al., *Association of Parent Training With Child Language Development A Systematic Review and Meta-analysis.* JAMA PEDIATRICS, 2019.
8.Dubin and Lieberman-Betz, *Naturalistic Interventions to Improve Prelinguistic Communication for Children with Autism Spectrum Disorder: a Systematic Review.* Review Journal of Autism and Developmental Disorders, 2020.
9.Novak and Morgan, *Chapter 23 - High-risk follow-up: Early intervention and rehabilitation*, in *Handb. Clin. Neurol.*, de Vries and Glass, Editors. 2019, Elsevier.
10.Rinaldi, et al., *Efficacy of the Treatment of Developmental Language Disorder: A Systematic Review.* Brain Sci., 2021.
11.Horiuchi, et al., *"Caregivers' awareness matters": Call for a child-centered approach to early detection of autism spectrum disorder through well-child visits in Japan.* Child Care Health Dev, 2023.
12.Sim, et al., *Predictive validity of preschool screening tools for language and behavioural difficulties: A PRISMA systematic review.* PLoS One, 2019.
13.Wetherby, et al., *Validation of the Infant—Toddler Checklist as a broadband screener for autism spectrum disorders from 9 to 24 months of age.* Autism, 2008.
14.Tell. *Swedish Child health services.*
15.Wallby and Hjern, *Child health care uptake among low-income and immigrant families in a Swedish county.* Acta Paediatr., 2011.
16.Westerlund, et al., *Can Severely Language Delayed 3-Year-Olds Be Identified at 18 Months? Evaluation of a Screening Version of the MacArthur-Bates Communicative Development Inventories.* J. Speech. Lang. Hear. Res., 2006.
17.Wetherby and Prizant, *Communication and Symbolic Behavior Scales Developmental Profile, First Normed Edition*. 2002,
18.Wetherby and Prizant, *Communication and Symbolic Behavior Scales Developmental Profile Infant/Toddler Checklist.* 2001.
19.Wetherby, et al., *Validity and reliability of the communication and symbolic behavior scales developmental profile with very young children.* J. Speech. Lang. Hear. Res., 2002.
20.Wetherby, et al., *Early identification of children with communication disorders: concurrent and predictive validity of the CSBS Developmental Profile.* Infants & Young Children: An Interdisciplinary Journal of Early Childhood Intervention, 2003.

Confidential: For Review Only

14

21.Borkhoff, et al., *Predictive validity of the Infant Toddler Checklist in primary care at the 18-month visit and developmental diagnosis at 3–5 years: a prospective cohort study.* BMJ Paediatrics Open, 2022.
22.Fäldt, et al., *Infant-Toddler Checklist identifies 18-month-old children with communication difficulties in the Swedish child healthcare setting.* Acta Paediatr., 2021.
23.Fäldt, et al., *Nurses' experiences of screening for communication difficulties at 18 months of age.* Acta Paediatr., 2019.
24.Woolf, *The meaning of translational research and why it matters.* Jama, 2008.
25.Rubenstein and Pugh, *Strategies for promoting organizational and practice change by advancing implementation research.* Journal of General Internal Medicine, 2006.
26.Glasgow, et al., *Evaluating the public health impact of health promotion interventions: the RE-AIM framework.* American journal of public health (1971), 1999.
27.Glasgow, et al., *RE-AIM Planning and Evaluation Framework: Adapting to New Science and Practice With a 20-Year Review.* Frontiers in Public Health, 2019.
28.DEMOS. *Segregationsbarometern [Segregation barometer].*
29.R Core Team, *R: A language and environment for statistical computing*. 2022, R Foundation for Statistical Computing: Vienna, Austria.
30.IBM Corp., *IBM SPSS Statistics for Macintosh*. 2022: Armonk, NY.
31.Damschroder, et al., *Fostering implementation of health services research findings into practice: a consolidated framework for advancing implementation science.* Implementation science : IS, 2009.
32.Guthrie, et al., *Accuracy of Autism Screening in a Large Pediatric Network.* Pediatrics, 2019.
33.Rudolph, *Case History Risk Factors for Specific Language Impairment: A Systematic Review and Meta-Analysis.* Am. J. Speech Lang. Pathol., 2017.
34.Nurse, et al., *Association Between Family Income and Positive Developmental Screening Using the Infant Toddler Checklist at the 18-Month Health Supervision Visit.* The Journal of Pediatrics, 2024.
35.Morgan, et al., *Cross-Cohort Evidence of Disparities in Service Receipt for Speech or Language Impairments.* Except. Child., 2017.
36.Morris, et al., *The answer is 17 years, what is the question: understanding time lags in translational research.* J. R. Soc. Med., 2011.
37.Wallis, et al., *Adherence to screening and referral guidelines for autism spectrum disorder in toddlers in pediatric primary care.* PLoS One, 2020.
38.Weitlauf, et al., *Black Families' Experiences of Developmental Screening: Review of Well-Child Visits to Inform Enhanced Autism Spectrum Disorder Risk Assessment.* J. Dev. Behav. Pediatr., 2022.
39.Marjomaa, *Lång kö till logoped för barn i Västra Götaland [Long waiting time to get SLT service for children in Västra Götaland]*, in *SVT Väst*. 2021: Göteborg.
40.Wilson and Jungner, *Principles and practice of screening for disease.* 1968, WHO: Geneva.

Confidential: For Review Only

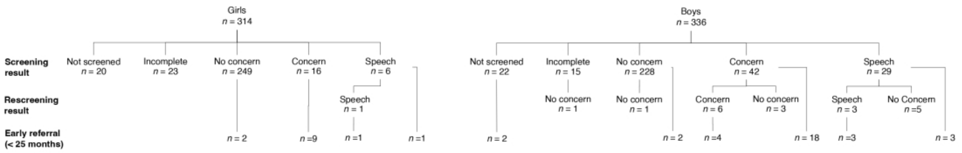

Figure 1 Screening results and referrals for girls and boys

546x134mm (150 x 150 DPI)

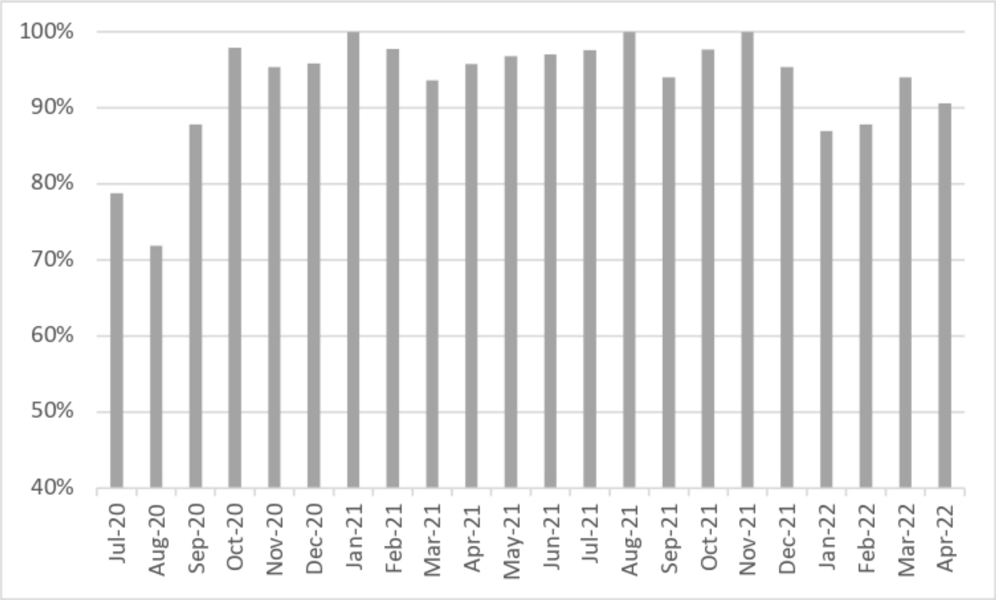

Figure 2 Screening rates across implementation time

127x76mm (150 x 150 DPI)
